# Supplementary material for: A Systems Biology Approach for Prioritizing ASD Genes in Large or Noisy Datasets
Source: Int J Mol Sci. 2025 Feb 27;26(5):2078. doi: 10.3390/ijms26052078 (PMC11900372; doi:10.3390/ijms26052078)
Supplement: Supplementary file 1 [file ijms-26-02078-s001.zip › Supplementary Figures.pdf]

SUPPLEMENTARY FIGURES

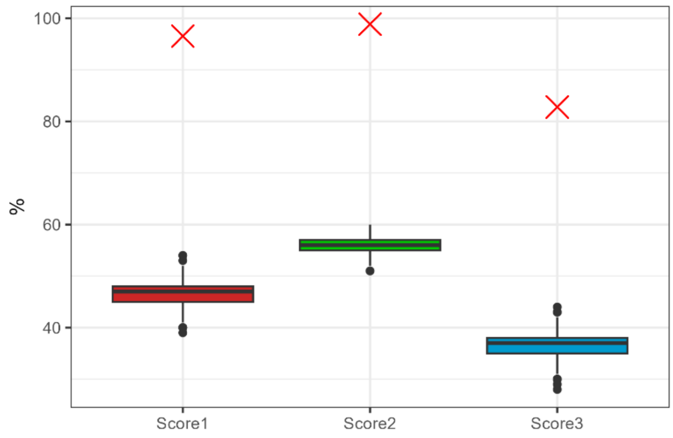

Figure S1. Boxplot showing the percentage distribution of gene scores (Score1, Score2, Score3) obtained from 1,000 Monte-Carlo iterations. The red crosses indicate the percentage values corresponding to network A for each score type. The plot illustrates the variability in the results of random sampling compared to the known values for the network.

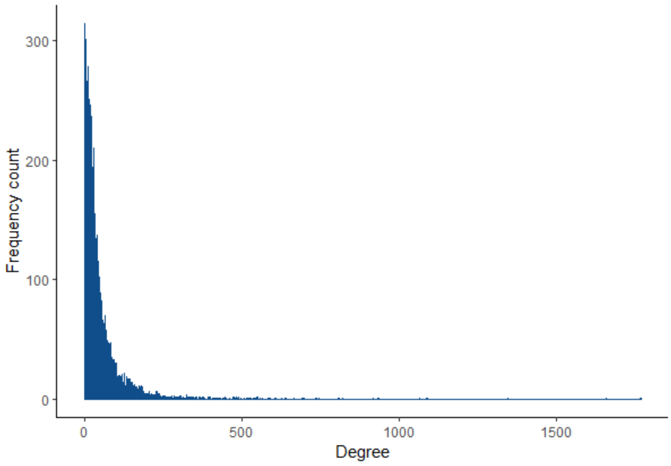

Figure S2. Histogram of the frequency count of nodes' degree in network A. The degree distribution follows a power law, typical of real networks. Most nodes exhibit a relatively small degree, while a few are highly connected to many other nodes, exhibiting higher degrees. This type of distribution is characteristic of undirected biological networks. In such networks, degree provides an immediate measure of a node's regulatory relevance, with proteins having high degree in signaling networks expected to function as central regulatory hubs.

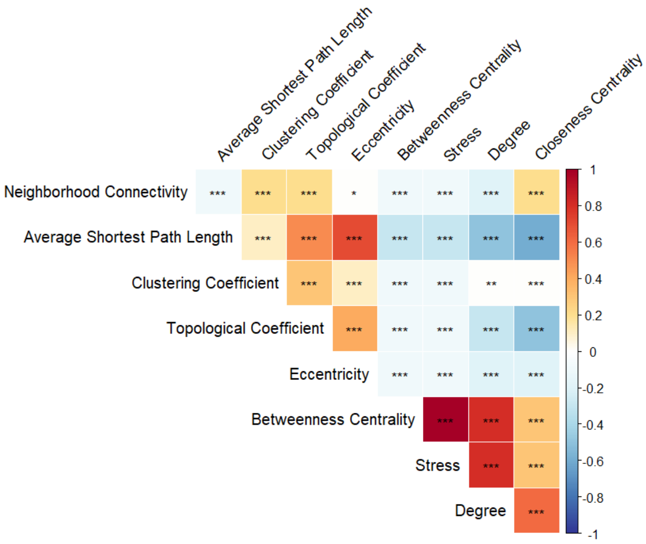

Figure S3. Correlation matrix of topological metrics in network A. Topological metrics such as average shortest path length, closeness centrality, clustering coefficient, eccentricity, radiality, and stress were compared. The color indicates the Pearson r-value, and significance levels are displayed: * for $p < 0.05$, ** for $p < 0.01$, *** for $p < 0.001$. The matrix shows that all metrics were mostly correlated, either positively or negatively, with betweenness centrality. Therefore, betweenness centrality was chosen to rank all protein-coding genes present in network A.
