# Supplementary material for: A Systems Biology Approach for Prioritizing ASD Genes in Large or Noisy Datasets
Source: Int J Mol Sci. 2025 Feb 27;26(5):2078. doi: 10.3390/ijms26052078 (PMC11900372; doi:10.3390/ijms26052078)
Supplement: Supplementary file 1 [file ijms-26-02078-s001.zip › Supplementary File S1.pdf]

The list of DECIPHER IDs for the 135 patients carrying the 177 CNVs classified as VUS and included in the validation cohort:

296418, 299790, 299791, 300043, 300108, 300160, 300164, 300209, 300770, 301271, 301274, 314280, 314644, 314659, 314813, 314859, 318799, 322825, 322830, 323448, 323462, 323517, 323522, 324401, 324409, 324416, 325643, 325643, 327249, 327258, 328072, 328488, 332442, 333221, 333223, 333223, 333230, 333235, 339137, 339433, 339760, 340337, 343922, 345525, 357454, 359652, 359722, 364034, 365858, 366365, 366396, 366429, 366491, 366498, 366500, 366510, 366567, 366575, 366576, 366636, 368550, 381257, 384423, 384424, 385941, 391510, 402073, 407756, 409177, 409388, 413603, 413993, 414042, 414043, 414047, 414109, 414111, 414113, 414114, 414115, 414116, 414118, 414119, 414120, 414121, 414122, 414123, 414124, 414125, 414126, 414127, 414128, 414129, 414130, 414131, 414132, 414133, 414135, 414136, 414248, 415025, 415035, 419509, 428832, 429231, 433541, 451532, 451533, 451534, 454296, 456693, 484698, 484700, 484980, 484981, 484983, 485057, 485059, 485102, 485105, 485107, 485108, 485136, 486579, 486580, 486614, 486615, 486617, 486619, 486781, 486782, 486783, 486785, 486786, 503057).
